# Exchange Factor EFA6R Requires C-terminal Targeting to the Plasma Membrane to Promote Cytoskeletal Rearrangement through the Activation of ADP-ribosylation Factor 6 (ARF6)*

**DOI:** 10.1074/jbc.M113.534156

**Published:** 2014-10-08

**Authors:** Venkateswarlu Kanamarlapudi

**Affiliations:** From the Institute of Life Science 1, College of Medicine, Swansea University, Singleton Park, Swansea SA2 8PP, United Kingdom

**Keywords:** Actin, Cytoskeleton, Guanine Nucleotide Exchange Factor (GEF), Ovarian Cancer, Plasma Membrane, ARF6, CC, EFA6R, PH, PIP_2_

## Abstract

ADP-ribosylation factor 6 (ARF6) small GTPase regulates membrane trafficking and cytoskeleton rearrangements at the plasma membrane (PM) by cycling between the GTP-bound active and GDP-bound inactive conformations. Guanine nucleotide exchange factors (GEFs) activate ARF6. The exchange factor for ARF6 (EFA6) R has been identified as a biomarker for ovarian cancer. EFA6R shares the catalytic Sec7, pleckstrin homology (PH), and coiled coil (CC) domains of the other EFA6 family GEFs. Here we report the functional characterization of EFA6R. Endogenous EFA6R was present in the plasma membrane fraction. The exogenously expressed FLAG- and GFP-tagged EFA6R were targeted to the PM. *In vitro*, GFP-EFA6R associated weakly but preferentially with phosphatidylinositol 4,5-bisphosphate (PIP_2_) through the PH domain. EFA6R required both its PH and CC domains localized at the C terminus to target the PM. Consistent with this, EFA6R lacking the CC domain (EFA6RΔCC) was released from the PM into the cytosol upon PIP_2_ depletion, whereas EFA6R release from the PM required both PIP_2_ depletion and actin destabilization. These results suggest that the dual targeting via the PH and CC domains is important for the PM localization of EFA6R. EFA6R specifically catalyzed the GTP loading of ARF6 in mammalian cells. Moreover, EFA6R regulated ARF6 localization and thereby actin stress fiber loss. The GEF activity of EFA6R was dependent on the presence of the Sec7 domain. The PH and CC domains were also required for the *in vivo* GEF activity of EFA6R but could be functionally replaced by the C*AAX* motif of K-Ras, suggesting a role for these domains in the membrane targeting of EFA6R.

## Teaser

**Background:** The subcellular localization and cellular functions of EFA6R are unknown.

**Results:** EFA6R requires dual targeting through the PH and CC domains to localize to the plasma membrane and function as a GEF.

**Conclusion:** Plasma membrane-localized EFA6R stimulates actin reorganization through ARF6 activation.

**Significance:** This study provides insight into the localization and cellular functions of EFA6R.

## Introduction

ADP-ribosylation factors (ARFs)^2^ are a family of Ras-related small GTPases that are expressed abundantly in all eukaryotic cells and some bacteria. Six ARF proteins (ARFs 1–6) are found in mammals, although ARF2 is absent in humans. ARFs 1–5 are mainly localized to the Golgi and involved in intracellular membrane trafficking (1). In contrast, ARF6 is localized to the plasma membrane (PM) and endosomes where it regulates membrane trafficking and actin cytoskeleton remodeling events that are required for a wide variety of cellular processes such as exocytosis, endocytosis, formation of neuronal axonal branches and dendritic spines, and cell motility (2–4). Like other small GTPases, ARFs act as binary switches by cycling between inactive GDP-bound and active GTP-bound forms. The active GTP-bound ARFs interact with downstream effectors to mediate various cellular responses. The active/inactive cycle of ARFs is tightly regulated by guanine nucleotide exchange factors (GEFs) and GTPase-activating proteins. GEFs activate ARFs by catalyzing the exchange of bound GDP for GTP. GTPase-activating proteins inactivate ARFs by stimulating their GTPase activity for the hydrolysis of bound GTP to GDP (5, 6).

ARF GEFs contain a conserved Sec7 catalytic domain, which has facilitated the identification of these ARF regulators in eukaryotes (7). There are more than 15 mammalian ARF GEFs, which have been classified into six families based on their sequence similarities (8). The ARF substrate specificity, localization, and function have not been determined for many GEFs. Because ARF1 and ARF6 have distinct subcellular localization, it is believed that the specificity of GEFs toward their substrate ARF isoform is dependent on their subcellular localization (9) with ARF1 GEFs being localized to the Golgi and ARF6 GEFs being found at the PM where GDP/GTP exchange on ARF6 has been shown to occur (10). Among the six families of ARF GEFs, the EFA6, cytohesin, and brefeldin A-resistant ARF GEF (BRAG) families of GEFs have been shown to activate ARF6 (5). However, the cytohesins can also activate ARF1 *in vitro* and *in vivo* (11, 12). In addition to the Sec7 domain, both EFA6 and cytohesin family members contain a phosphatidylinositol (PI)-binding pleckstrin homology (PH) domain and one or more coiled coil (CC) domains, which may be important for protein-protein interactions and in the case of the ARF6 GEF EFA6 is involved in cytoskeletal rearrangements (13–15).

The EFA6 family comprises four mammalian members (EFA6A–C and R), which show distinct tissue distribution at the mRNA level. EFA6A and EFA6C are expressed predominantly in the brain, whereas EFA6B and EFA6R (also known as EFA6D/HCA67/PSD3) are more widely expressed (16–18). Although EFA6B mRNA is not expressed in the brain, EFA6A, EFA6C, and EFA6R have been shown to exhibit distinct regional distributions in the brain (18). These expression profile studies suggest that each member of the EFA6 family may have diverse physiological functions in different tissues. EFA6R was originally identified by immunological screening of recombinant cDNA libraries obtained from hepatocellular carcinoma samples and has since been shown to be up-regulated in colon cancer and down-regulated in breast, prostate, glioblastoma, and ovarian cancer (7, 14, 19–22). Moreover, analysis of genes with extremely imbalanced expression in the chromosomal regions associated with breast cancer metastasis has identified EFA6R as a candidate metastasis suppressor gene (19).

Here we report further characterization of EFA6R by analyses of its protein expression in various tissues and cell lines, subcellular localization, ARF substrate specificity, and cellular functions. We show that EFA6R was expressed ubiquitously and functioned as a GEF for ARF6, but not ARF1, *in vivo*, and that it was targeted to the PM via binding to PIP_2_ directly and actin indirectly through its PH and CC domains, respectively. We also show that EFA6R was involved in reorganization of the actin cytoskeleton by activating ARF6.

## EXPERIMENTAL PROCEDURES

### 

#### 

##### Materials

The primary antibodies used were mouse anti-HA HA11 (Covance), mouse anti-GFP (Roche Applied Science), mouse anti-FLAG M2 (Sigma), mouse anti-α-tubulin (Sigma), and mouse anti-epidermal growth factor receptor (Santa Cruz Biotechnology). Rabbit anti-EFA6R antibody was produced against a peptide representing the last 15 C-terminal amino acids of EFA6R and affinity-purified by using the immunizing peptide-coupled resin through a commercial vendor (Eurogentec). Cy3- and Cy5-conjugated anti-mouse IgG (Jackson ImmunoResearch Laboratories) secondary antibodies and TRITC-conjugated phalloidin (Sigma) were used for immunofluorescence. Horseradish peroxidase-conjugated anti-mouse and anti-rabbit IgG (GE Healthcare) secondary antibodies were used for immunoblotting. INSTA-Blot human tissues and Mowiol were from Merck Millipore. All other chemicals were from Sigma unless otherwise specified.

##### Plasmids

The full-length human EFA6R (isoform B) and its deletion mutant cDNAs were amplified from mammalian gene collection clone 104121 (Source Bioscience) by PCR using High Fidelity *Taq* DNA polymerase (Roche Applied Science) and sequence-specific primers containing EcoRI (sense) and SalI (antisense) restriction sites. The cDNA was digested with EcoRI and SalI and cloned into the same sites of pCMV-Tag2b (Stratagene) for expression as FLAG-tagged fusion protein and pEGFPC2 vector (Clontech) for expression as green fluorescent protein (GFP)-tagged fusion protein in mammalian cells. The EFA6R_E134K_ mutant was generated using a QuikChange II site-directed mutagenesis kit (Stratagene). The GFP-EFA6R and its deletion mutants were targeted to membranes by attaching a C-terminal C*AAX* motif using PCR with a 3′-primer containing the coding sequence for the C*AAX* motif from K-Ras (KDGKKKKKKSKTKCVIM) as described previously (23). The ARF6-HA/pXS, ARF1-HA/pXS, ARF6_T27N_-HA/pXS, and ARF1_T31N_-HA/pXS constructs were used to express ARF proteins with a C-terminal hemagglutinin (HA) epitope tag in mammalian cells (24). ARF6_T44N_-HA/pXS, GST-GGA3 protein binding domain (PBD; amino acids 1–316), FLAG-EFA6A, and GFP-EFA6A constructs have been described previously (25–28). EFA6C cDNA was obtained by PCR amplification of mammalian gene collection clone 45998, digested with EcoRI (sense) and SalI (antisense), and cloned into the same sites of pEGFP-C2 (Clontech) to obtain GFP-EFA6C construct. The PLCδ1PH-GFP, mRFP-FKBP-5-ptase-dom (the 5-phosphatase domain of human type IV 5-phosphatase enzyme fused to FK506-binding protein 12-mRFP), and PM-FRB-CFP (the PM-targeted FKBP12-rapamycin binding-CFP) constructs, kindly provided by Dr. Tamas Balla (National Institutes of Health), have been described previously (29).

##### siRNA Oligonucleotides

Human EFA6R siRNA1 (5′-GCUACUGAGUAACGAUGAA-3′) and siRNA2 (5′-GGAGAAAGCUAACGGAACA-3′) duplexes were used. As a negative control, an siRNA duplex consisting of a unique sequence that does not have significant homology to any mammalian gene sequences was used. Human ARF6 siRNA has been described previously (27). The 21-nucleotide siRNA duplexes were synthesized by Eurogentec.

##### Cell Culture and Transfection

HeLa and COS7 cells were maintained in DMEM supplemented with 10% fetal calf serum and antibiotics in a 37 °C, 5% CO_2_ incubator. Cells were transfected with plasmids for 2 days using Genejuice (Novagen) or siRNA for 3 days using INTERFERin (Polyplus Transfection) according to the manufacturers' instructions. For immunofluorescence, cells were seeded onto sterile 13-mm glass coverslips in 24-well plates 24 h before transfection. For ARF activation assays, cells were seeded into either 6- or 10-cm tissue culture dishes 24 h before transfection.

##### Cell Fractionation

Cells were harvested from a 10-cm plate by scraping in 1 ml of ice-cold resuspension buffer (10 mm Tris-HCl, pH 7.5, and 0.1 m sucrose with 1% mammalian protease inhibitor mixture) and lysed by passing 5–10 times through a 26-gauge needle attached to a 1-ml syringe (30). Cell lysate was centrifuged at 1000 × *g* for 5 min at 4 °C to obtain the postnuclear supernatant. The postnuclear supernatant was centrifuged at 100,000 × *g* for 30 min at 4 °C to obtain the cytosolic (supernatant) and membrane fractions (pellet). The protein concentration of the cytosolic and membrane fractions was determined using the bicinchoninic acid (BCA) method. Whole cell extracts and cytosolic and membrane fractions were subjected to immunoblotting to assess the levels of α-tubulin (a cytosolic protein), epidermal growth factor receptor (a membrane protein), and EFA6R in each fraction.

The plasma membrane fraction was prepared from HeLa cells as described previously (31). Briefly, HeLa cells were incubated with 0.1 mg/ml EZ-Link Sulfo-NHS-SS-Biotin (Pierce) for 15 min for cell surface biotinylation. Cells were then scraped in ice-cold hypotonic buffer (10 mm HEPES, pH 7.5, 1.5 mm MgCl_2_, and 10 mm KCl with 1% mammalian protease inhibitor mixture) and lysed by using Dounce homogenization. Cell lysates were centrifuged at 1000 × *g* for 5 min at 4 °C to obtain the postnuclear supernatant. After adjusting the KCl concentration to 150 mm, the postnuclear supernatant was incubated with streptavidin magnetic beads (Pierce) for 1 h at 4 °C. The beads were washed with hypotonic buffer to obtain the plasma membrane fraction or 1 m KCl and 0.1 m Na_2_CO_3_, pH 11.5 and then hypotonic buffer to obtain the integral plasma membrane fraction.

##### Immunoblotting

Protein samples were separated by SDS-PAGE and transferred onto PVDF membrane (Whatman). Blots were probed with primary antibody followed by the appropriate HRP-conjugated secondary antibody and developed using ECL substrate (28, 32).

##### In Vitro PI Binding Assay

The *in vitro* PI binding assay was performed as described previously (23). Briefly, COS7 cells transfected for 2 days with GFP, GFP-tagged EFA6R or its mutants, or GFP-PLCδPH were treated with lysis buffer (10 mm HEPES, pH 7.4, 150 mm NaCl, 0.5% Nonidet P-40, and 5 mm dithiothreitol) containing 1% mammalian protease inhibitor mixture. Cell lysates were incubated with avidin beads (Pierce) or avidin beads coupled to biotinylated PI (Cell Signaling Technology) for 2 h at 4 °C. For competition binding experiments, biotinylated PI 3,4-P_2_ beads were incubated with the cell lysate in the presence of 1 nm to 10 μm non-biotinylated water-soluble (diC_8_) PIs (Cell Signaling Technology). Beads were washed twice with lysis buffer, and bound proteins were eluted by boiling in SDS-PAGE sample buffer. Proteins were then separated by SDS-PAGE and transferred to PVDF membrane. The blots were probed with an anti-GFP antibody, and the relative intensity of detected bands was determined by scanning and analyzing using ImageJ software.

##### GST-GGA3 PBD Pulldown Assay

ARF6 activation was assessed by using the GST-GGA3 PBD pulldown assay as described previously (33). After transfection for 48 h, cells were serum-starved for 2 h and lysed in ice-cold modified radioimmune precipitation assay buffer (50 mm Tris, pH 7.5, 150 mm NaCl, 10 mm MgCl_2_, 1% Triton X-100, 0.5% sodium deoxycholate, 0.1% SDS, and 1% mammalian protease inhibitor mixture). Lysates were incubated for 2 h at 4 °C with 10 μl of GST-GGA3 PBD beads, which were prepared as described previously (27, 28). The beads were washed three times with 1 ml of wash buffer (50 mm Tris, pH 7.5, 150 mm NaCl, 10 mm MgCl_2_, and 1% Triton X-100). Lysates not incubated with beads were used as input controls. HA-tagged ARF6- or ARF1-GTP bound to the beads and total ARF1 or ARF6 in the inputs were determined by immunoblotting using an anti-HA antibody. GFP, GFP-EFA6R, and GFP-EFA6A expression in the inputs was assessed by immunoblotting using an anti-GFP antibody. Immunoblots were scanned, and the GTP-bound ARF6 precipitated with GST-GGA3 PBD beads was normalized to total ARF6 levels in the lysates to compare ARF6-GTP levels under different conditions (34).

##### Ionomycin and Rapamycin Treatments

2 days after transfection, cells were washed three times with PBS and stimulated with either 10 μm ionomycin (Tocris) for 5 min in Hanks' balanced salt solution or 100 nm rapamycin (LC Laboratories) for 3 min in PBS at 37 °C. Where indicated, the cells were treated with cytochalasin D at 10 μg/ml for 30 min before exposing to ionomycin or rapamycin (30).

##### Immunofluorescence

Immunofluorescence was carried out as described previously (35). Briefly, 2 days after transfection, cells were serum-starved for 2 h and then fixed with 4% paraformaldehyde for 15 min. Where required, cells were permeabilized with 0.2% Triton X-100 in PBS for 10 min, blocked in blocking buffer (1% BSA and 0.1% Triton X-100 in PBS) for 30 min, and then incubated with an anti-HA mouse monoclonal antibody diluted (1:500) in blocking buffer for 1 h. Cells were washed three times with wash buffer (0.1% Triton X-100 in PBS) and incubated in blocking buffer containing a 1:500 dilution of Cy3-conjugated anti-mouse antibody, 1:5000 dilution of TRITC-phalloidin, or 1:500 dilution of Cy5-conjugated anti-mouse antibody and 1:5000 dilution of TRITC-phalloidin for 1 h. Cells were then washed three times with wash buffer, and the coverslips were mounted on glass microscopic slides with mounting solution (0.1 m Tris-HCl, pH 8.5, 10% Mowiol, and 50% glycerol) containing 2.5% 1,4-diazabicyclo[2.2.2.]octane. Immunofluorescence staining was visualized using a Zeiss LSM710 confocal microscope. For quantification, the densitometric ratio of plasma membrane-localized EFA6R *versus* total EFA6R was calculated using ImageJ software.

##### Data Analysis

The ImageJ program was used for densitometric analysis. Data were analyzed using the GraphPad Prism program. Results are expressed as the mean ± S.D. of three experiments. Statistical significance was determined by one-way or two-way analysis of variance where *p* > 0.05 was considered as statistically not significant and *p* < 0.01 (**) and *p* < 0.001 (***) were considered as statistically significant. Confocal images are representative of 190–200 transfected cells from three independent experiments. Immunoblotting data are representative of three separate experiments.

## RESULTS

### 

#### 

##### Domain Structure and Expression of EFA6R

**FIGURE 1.**
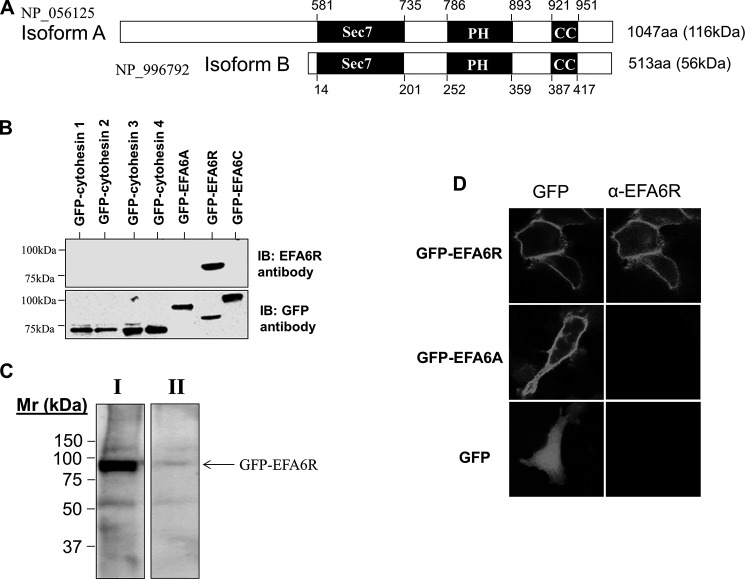
**EFA6R domain structure and its antibody characterization.** *A*, schematic representation of the domain structure of isoforms A and B of EFA6R. *Sec7*, Sec7 domain; *PH*, PH domain; *CC*, coiled coil motif; *aa*, amino acids. *B*, immunoblot (*IB*) analysis of the specificity of an anti-peptide EFA6R rabbit polyclonal antibody. The lysates (5 μg of protein/lane) of COS7 cells expressing GFP-tagged cytohesins 1–4, EFA6A, EFA6B, and EFA6R were separated by SDS-PAGE, blotted onto PVDF membrane, and probed with an anti-GFP or an anti-EFA6R antibody. *C*, immunoblot (*IB*) analysis of the specificity of EFA6R antibody using the immunizing peptide. The lysates (5 μg of protein/lane) of COS7 cells expressing GFP-EFA6R were separated by SDS-PAGE, blotted onto PVDF membrane, and probed with the anti-EFA6R antibody (*lane I*) or the anti-EFA6R antibody preincubated with the immunizing peptide (*lane II*). *D*, immunostaining of HeLa cells transfected with GFP, GFP-EFA6R, or GFP-EFA6A using the anti-EFA6R antibody. HeLa cells transfected with GFP or the GFP-EFA6R or GFP-EFA6A construct for 2 days were fixed, stained with the anti-EFA6R antibody, and visualized by a confocal microscopy.

EFA6R shares 59% amino acid identity to EFA6A and contains the same domain structure as EFA6A: an N-terminal variable region followed by a Sec7 domain, a PH domain, and a C-terminal CC domain. In the Sec7 domain, EFA6R shows high identity to EFA6A (66%), EFA6B (60%), and EFA6C (70%) (16). The human gene for EFA6R is located on chromosome 8, position 8p22 (14). Two splice variants (isoforms A and B) have so far been predicted through alternative splicing of *EFA6R* that differ from each other in length of the N-terminal variable region (Fig. 1*A*). However, the EFA6R isoform A domain structure is identical to that of isoform B. RT-PCR analysis by Sakagami *et al.* (18) revealed that EFA6R mRNA is expressed ubiquitously in mouse tissues, and therefore, we assessed EFA6R protein expression in various human tissues and cell lines by immunoblotting. For this purpose, a rabbit polyclonal antibody was raised against the last 15 C-terminal residues of EFA6R.

To verify antibody specificity, we performed immunoblot analysis of extracts from cells overexpressing cytohesins 1–4, EFA6A, EFA6C, and EFA6R fused with an N-terminal GFP tag (Fig. 1*B*). This analysis confirmed that the EFA6R anti-peptide antibody recognized an ∼85-kDa protein only in cells expressing GFP-EFA6R. Cytohesins 1–4, EFA6A, and EFA6C were not recognized by the EFA6R antibody, whereas an anti-GFP antibody detected all of the GFP-tagged ARF GEFs (indicating the expression of all ARF GEFs). However, the 85-kDa protein was not detected by anti-EFA6R antibody that had been preincubated with the immunizing peptide (Fig. 1*C*). We also analyzed whether the EFA6R antibody labels cells overexpressing GFP-tagged EFA6R or EFA6A and compared the pattern of staining with that observed with GFP fluorescence. In cells expressing GFP-EFA6R, the EFA6R antibody staining pattern was identical to that observed with the GFP fluorescence (Fig. 1*D*). However, in cells expressing GFP-EFA6A, anti-EFA6R antibody did not immunostain transfected cells (Fig. 1*D*), suggesting that, although specific for EFA6R, the EFA6R antibody is incapable of recognizing (low level) endogenous EFA6R by immunostaining.

**FIGURE 2.**
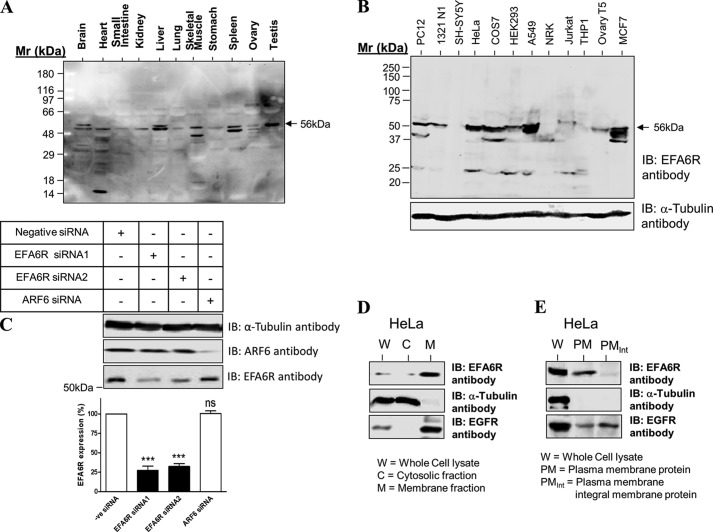
**Analysis of EFA6R protein expression in various tissues, cell lines, and cell fractions.** EFA6R protein expression in human tissues (*A*), cell lines (*B*), cell fractions (*D*), and plasma membrane proteins (*E*) was analyzed by immunoblotting (*IB*). HeLa cells were mechanically disrupted and fractionated into cytosolic and membrane fractions by ultracentrifugation. The plasma membrane proteins were isolated by disrupting cell surface-biotinylated HeLa cells and incubating the lysates with streptavidin beads. The beads were washed with high salt and high pH buffers to obtain integral plasma membrane proteins. Tissue (50 μg of protein/lane) and whole cell lysates (100 μg of protein/lane) or the cytosolic and the membrane fractions (20 μg of protein/lane) were separated by SDS-PAGE, blotted onto PVDF membrane, and probed with an anti-EFA6R antibody. HeLa whole cell lysate, the cytosolic and membrane fractions, and the plasma membrane proteins were also immunoblotted for the presence of the cytosolic protein α-tubulin and the membrane protein epidermal growth factor receptor (*EGFR*). The lysates of different cell lines were probed with an anti-α-tubulin antibody to ensure equal loading. *C*, *upper panel*, EFA6R protein expression significantly reduced by siRNA-mediated down-regulation of EFA6R. HeLa cells were transfected with negative (*-ve*) control siRNA, EFA6R siRNA1, EFA6 siRNA2, or ARF6 siRNA (positive control). After 3 days, cells were lysed and immunoblotted (*IB*) with an anti-EFA6R rabbit polyclonal antibody, an anti-ARF6 mouse monoclonal antibody, or an anti-α-tubulin mouse monoclonal antibody. *Lower panel*, corresponding quantitative data for the *upper panel*. ***, *p* < 0.001; *ns*, not significant. *Error bars* represent S.D.

Having determined antibody specificity, EFA6R expression was analyzed in human cells and tissues by immunoblotting (Fig. 2, *A* and *B*). EFA6R was variably expressed in most tissues and cell lines tested where it was detected predominantly as a 56-kDa protein (isoform B). This EFA6R isoform B expression was abundant in liver, spleen, and testis; moderate in brain, heart, and skeletal muscle; and low in small intestine, kidney, lung, stomach, and ovary. Similarly, a major 56-kDa protein band was detectable at relatively high levels in HeLa, COS7, A549, and HEK293 cells. Moderate levels were expressed in the other cell lines tested except in human neuroblastoma (SH-SY5Y) and leukemic monocyte (THP-1) cell lines where staining was undetectable. These findings are consistent with observations that EFA6R is down-regulated in some cancers such as ovarian cancer and glioblastoma (14, 21). Furthermore, the 56-kDa band intensity was significantly reduced in the lysates of cells transfected with EFA6R siRNA1 or siRNA2 but not with either ARF6 siRNA (positive control) or universal negative control siRNA (Fig. 2*C*), which confirms the expression of EFA6R as a 56-kDa protein (isoform B).

To determine whether endogenous EFA6R was membrane-bound in HeLa cells, we mechanically disrupted the cells and assessed EFA6R expression in the cytosolic and membrane fractions by immunoblotting. Indeed, over 90% of the EFA6R was in the membrane fraction (Fig. 2*D*). However, the EFA6R associated with the membrane fraction was incompletely solubilized by treatment with 1% Triton-X-100, suggesting that it may associate with the Triton-insoluble cytoskeleton (not shown; see Fig. 5). Further analysis revealed the presence of endogenous EFA6R in the plasma membrane fraction of HeLa cells as non-integral membrane protein (Fig. 2*E*).

##### EFA6R Localizes to the Plasma Membrane

**FIGURE 3.**
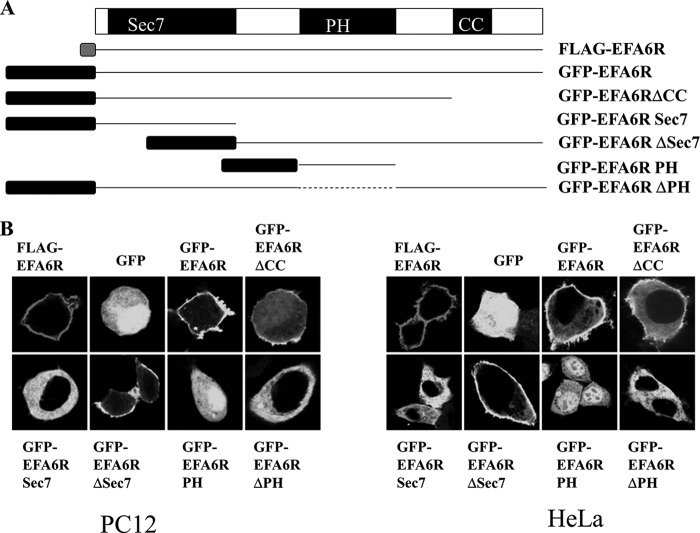
**EFA6R constructs and their subcellular localization.** *A*, a schematic representation showing the domain structure of the full-length EFA6R isoform B and the various deletion constructs used in this study. *B*, the intracellular localization of EFA6R expression constructs. HeLa and PC12 cells transfected with FLAG- or GFP-tagged EFA6R constructs were fixed, and FLAG-EFA6R-expressing cells were stained with an anti-FLAG antibody and visualized by confocal microscopy. The images are representative of 90–95% of 190–200 transfected cells from three different cell preparations.

Previous studies indicated that EFA6A localizes to the PM to activate ARF6 (15). Because EFA6R is structurally related to EFA6A and is expressed in the plasma membrane fraction, we assessed whether EFA6R also localizes to the PM in intact cells. For this purpose, GFP-tagged EFA6R was expressed in HeLa or PC12 cells, and subcellular localization was analyzed by confocal microscopy. GFP-tagged EFA6R was localized at the PM in both the cell lines tested. Like GFP-EFA6R, FLAG-tagged EFA6R was also localized at the PM, indicating that the large GFP tag had no effect on subcellular localization of EFA6R (Fig. 3). Furthermore, GFP-EFA6R showed the PM localization even when expressed at very low levels (data not shown). This together with the presence of endogenous EFA6R in the PM fraction indicates that EFA6R localization to the PM is not dependent on its expression levels. To examine the region(s) of the protein required for targeting EFA6R to the PM, we generated a series of deletion mutants of EFA6R with an N-terminal GFP fusion and analyzed their subcellular localization in HeLa and PC12 cells (Fig. 3). All of the fusion constructs containing both the PH and CC domains were able to localize to the PM. In addition, EFA6R lacking the CC domain (EFA6RΔCC) showed weak localization to the PM, whereas EFA6R without the PH domain (EFA6RΔPH) showed a diffuse cytoplasmic localization in both the cell lines. These results demonstrate that the PH and CC domains, but not the Sec7 domain, of EFA6R are required for its localization to the PM. Moreover, these findings suggest that the PH domain is necessary but not sufficient for the PM targeting of EFA6R.

##### EFA6R Binds PIP_2_ through Its PH Domain

**FIGURE 4.**
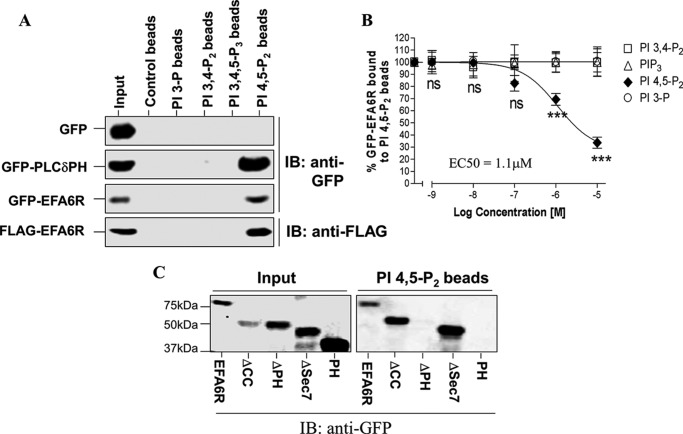
**EFA6R binds specifically to PI 4,5-P_2_ through the PH domain *in vitro*.** COS7 cells were transiently transfected with GFP, GFP-PLCδPH, FLAG-EFA6R, GFP-EFA6, or its deletion mutants. After 2 days, cells were lysed, and lysates were incubated with the indicated biotinylated PI lipids coupled to avidin beads either in the presence (*B*) or absence (*A* and *C*) of water-soluble non-biotinylated PI lipids for 2 h at 4 °C. Protein that remained bound to the PI beads after washing with 0.5% Nonidet P-40 was analyzed by immunoblotting (*IB*) with an anti-GFP or anti-FLAG antibody. *3-P*, 3-phosphate. ***, *p* < 0.001; *ns*, not significant. *Error bars* represent S.D.

Because PH domains are known to target proteins to the membrane by binding to PIs, we assessed whether the PH domain of EFA6R can bind to PIs and target EFA6R to the PM. To investigate whether EFA6R binds to PIs, we incubated the lysate of cells expressing either GFP (negative control), GFP-PLCδPH (positive control), GFP-EFA6R, or FLAG-EFA6R with avidin beads alone (control beads) or coupled to biotinylated PI 3,4,5-trisphosphate, PI 3,4-P_2_, PI 3-phosphate, or PI 4,5-P_2_. Like GFP-PLCδPH, GFP-EFA6R and FLAG-EFA6R selectively bound to the PI 4,5-P_2_-containing beads but not to the control beads or the beads that contained other PIs (Fig. 4*A*). However, GFP alone failed to bind any of the PIs tested under these conditions. These data suggest that EFA6R specifically interacts with PI 4,5-P_2_.

We next analyzed the sensitivity of EFA6R binding with PI 4,5-P_2_ by conducting competition binding experiments (Fig. 4*B*). PI 3-phosphate, PI 3,4-P_2_, and PI 3,4,5-trisphosphate failed to block EFA6R binding to PI 4,5-P_2_ beads at a very high concentration (10 μm). However, PI 4,5-P_2_ blocked EFA6R interaction with PI 4,5-P_2_ beads. The concentration required to block 50% of the binding (EC_50_) of PI 4,5-P_2_, PI 3,4-P_2_, PI 3,4,5-trisphosphate, and PI 3-phosphate was calculated from the displacement curve (Fig. 4*B*) as 1.1, >10, >10, and >10 μm, respectively. The ability of GFP-tagged EFA6R deletion mutants to bind PI was further analyzed to determine which domain is required for the binding to PI 4,5-P_2_ (Fig. 4*C*). Binding of the ΔSec7 and ΔCC mutants to PI 4,5-P_2_ beads was identical to that of the wild-type (WT) protein, whereas the ΔPH and PH mutants of EFA6R failed to bind PI 4,5-P_2_ beads under the same conditions. These results indicate that EFA6R requires the PH domain to interact with PI 4,5-P_2_. However, the PH domain alone failed to bind PI 4,5-P_2_ beads, which could be due to its improper folding. This is not uncommon because many PI-binding PH domains such as Bruton tyrosine kinase PH, cytohesin 1 PH, and cytohesin 2 PH domains require the adjacent sequences for proper folding and binding to PIs (36–38).

##### EFA6 Plasma Membrane Localization Is Dependent on Both the PH and CC Domains

**FIGURE 5.**
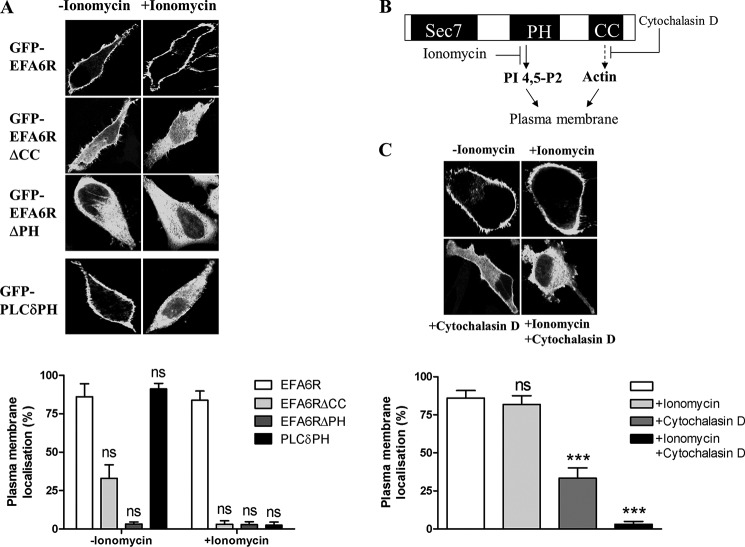
**Requirement of the PH and CC domains for membrane localization of EFA6R.** *A*, *upper panel*, HeLa cells transfected with GFP-tagged EFA6R constructs were treated with ionomycin. *Lower panel*, quantification of the ratio of plasma membrane EFA6R to total EFA6R by densitometric analysis. *B*, the effect of ionomycin and cytochalasin D on subcellular localization is shown schematically. *C*, *upper panel*, HeLa cells transfected with GFP-EFA6R were treated with ionomycin, cytochalasin D, or both and fixed, and subcellular localization was visualized by confocal microscopy. *Lower panel*, quantification of the ratio of plasma membrane-localized EFA6R to total EFA6R by densitometric analysis. ***, *p* < 0.001; *ns*, not significant. *Error bars* represent S.D.

To determine whether binding of EFA6R PH domain to PI 4,5-P_2_ is sufficient for the PM localization of EFA6R in intact cells, HeLa cells expressing GFP-tagged EFA6R, EFA6RΔPH, EFA6RΔCC, or PLCδPH were stimulated with ionomycin to cause the hydrolysis of PI 4,5-P_2_. Subcellular localization of proteins was then analyzed by confocal microscopy (39). The loss of PI 4,5-P_2_ caused EFA6RΔCC and PLCδPH to relocate from the PM to the cytosol, whereas EFA6R and EFA6RΔPH remained at the PM and cytosol, respectively (Fig. 5). When cells were treated with the actin-destabilizing chemical cytochalasin D, EFA6R showed weak localization to the PM, a localization pattern similar to that of EFA6RΔCC in untreated cells. Moreover, the loss of PI 4,5-P_2_ caused EFA6R to relocate from the PM to the cytosol in cytochalasin D-treated cells. Together these data suggest that EFA6R localizes to the PM by binding to PI 4,5-P_2_ directly through its PH domain and that the localization is further stabilized by the presence of an intact actin cytoskeleton and the CC domain of EFA6R.

**FIGURE 6.**
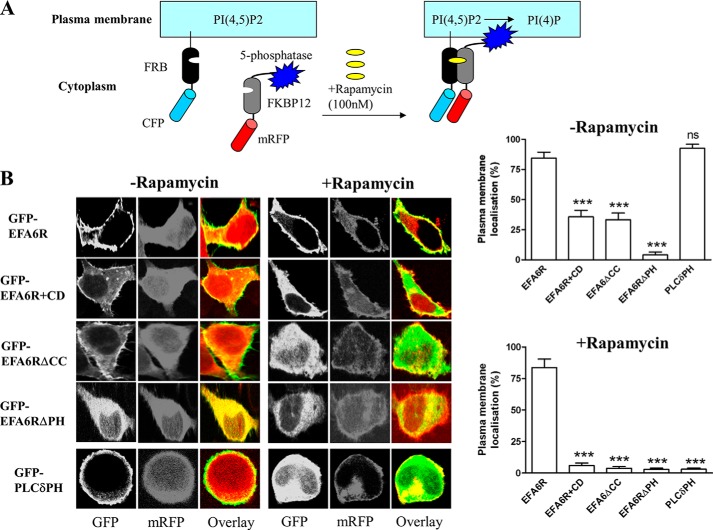
**Effect of the depletion of PIP_2_ by rapamycin-induced membrane targeting of the 5-phosphatase on EFA6R subcellular localization.** *A*, schematic of the 5-phosphatase and FRB constructs and the mechanism of PIP_2_ hydrolysis (redrawn from Varnai *et al.* (29)). *B*, *left panel*, HeLa cells were transfected with GFP-tagged EFA6R, EFA6RΔCC, EFA6RΔPH, or PLCδPH along with the membrane-targeted FRB-CFP and the mRFP-FKBP-5-phosphatase constructs. After 2 days, the cells were treated with cytochalasin D (*CD*), rapamycin, or both and fixed, and subcellular localization was visualized by confocal microscopy. *Right panel*, quantification of the ratio of plasma membrane EFA6R to total EFA6R by densitometric analysis. ***, *p* < 0.001; *ns*, not significant. *Error bars* represent S.D.

To provide direct support for a role of PI 4,5-P_2_ in EFA6R targeting to the PM, we analyzed the effect of depletion of PI 4,5-P_2_ using a rapamycin-inducible PI 4,5-P_2_ 5-phosphatase system on the EFA6R localization (Fig. 6). The basis of this system is that rapamycin induces translocation of the phosphatase domain of a PI 4,5-P_2_ 5-phosphatase to the PM (through heterodimerization of the PM-targeted FRB domain of mammalian target of rapamycin with FKBP12 fused to the cytosolic 5-phosphatase) causing depletion of PI 4,5-P_2_ (Fig. 6*A*). This system has been used previously to analyze the effect of PI 4,5-P_2_ depletion on the activity of TRPM8 and TRPV6 channels (29, 40). Rapamycin induced translocation of the fusion protein containing 5-phosphatase to the PM and relocation of PLCδPH (positive control) and EFA6RΔCC, but not EFA6R, from the PM to the cytosol (Fig. 6*B*). However, rapamycin induced EFA6R relocation to the cytosol in cytochalasin D-treated cells. We used EFA6RΔPH localized to the cytosol in the presence or absence of rapamycin as a negative control in the assay.

##### EFA6R Functions as an ARF6-specific GEF

**FIGURE 7.**
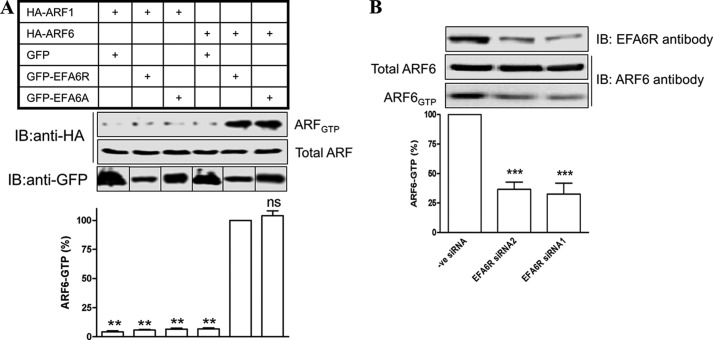
**EFA6R specifically acts as a GEF for ARF6 *in vivo*.** *A*, EFA6R activates ARF6, but not ARF1, in intact cells. COS7 cells were transfected with either ARF1-HA or ARF6-HA and either GFP-EFA6R, GFP-EFA6A, or GFP. After 2 days, cells were serum-starved and lysed, and GTP-bound ARF proteins were precipitated from the lysates with GST-GGA3 PBD beads. *Upper panel*, the GST-GGA3 PBD pulldowns (active GTP-bound ARF) and lysates (total ARF) were analyzed by immunoblotting (*IB*) with an anti-HA antibody. *Lower panel*, corresponding quantitative data for the *upper panel. B*, EFA6R down-regulation reduces endogenous ARF6 activation. HeLa cells transfected with negative (*-ve*) control siRNA, EFA6R siRNA1, or EFA6 siRNA2 for 3 days were lysed, and the GTP-bound endogenous ARF proteins were precipitated from the lysates with GST-GGA3 PBD beads. *Upper panel*, the GST-GGA3 PBD pulldowns (active GTP-bound ARF6) and lysates (total ARF6 and EFA6R) were analyzed by immunoblotting (*IB*) with ARF6 and EFA6R antibodies. *Lower panel*, corresponding quantitative data for the *upper panel*. **, *p* < 0.01; ***, *p* < 0.001; *ns*, not significant. *Error bars* represent S.D.

To determine whether or not EFA6R could function as an ARF GEF in intact cells, we co-expressed either GFP-EFA6R, GFP-EFA6, or GFP with either HA-ARF1 or HA-ARF6 in COS7 cells and subjected the lysates to a pulldown assay with the VHS-GAT domain of the ARF effector GGA3 (the GST-GGA3 PBD assay), which has been shown previously to bind exclusively to the GTP-bound form of ARFs (41). Western blotting with an anti-HA antibody revealed expression of ARF1 or ARF6 in the lysates of all transfected cells, whereas the GTP-bound form of ARF6 could only be detected in cells transfected with ARF6 and either EFA6R or EFA6, suggesting that EFA6R functions as a GEF selectively for ARF6 (Fig. 7*A*). Consistent with this, endogenous ARF6-GTP levels were reduced by siRNA-mediated knockdown of EFA6R (Fig. 7*B*).

##### The ARF6 GEF Activity of EFA6R Is Dependent on the Sec7 Domain and the Plasma Membrane Localization

**FIGURE 8.**
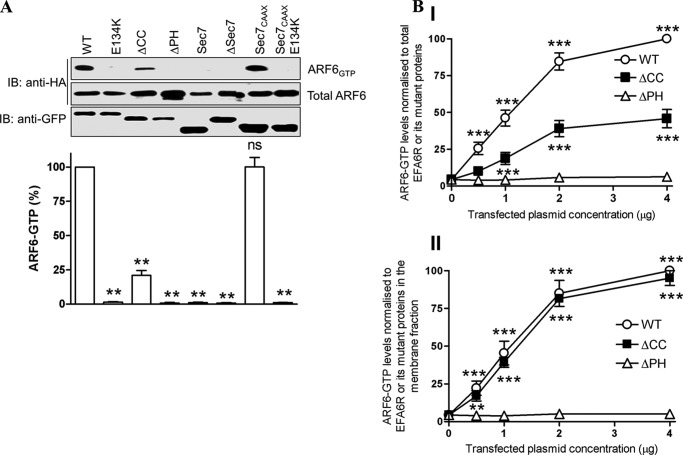
**EFA6R activates ARF6 in a functional Sec7 domain- and membrane localization-dependent manner.** *A*, EFA6R is dependent on its functional Sec7 domain and membrane localization for the ARF6 GEF activity. *Upper panel*, EFA6R WT, the E134K mutant, and the deletion constructs were expressed individually in COS7 cells with ARF6-HA, and the GEF activity of EFA6R was analyzed by pulldown of GTP-bound ARF6-HA. The GST-GGA3 PBD pulldowns (active ARF6) and lysates (total ARF6) were analyzed by immunoblotting (*IB*) with an anti-HA antibody. *Lower panel*, corresponding quantitative data for the *upper panel. B*, concentration dependent activation of ARF6 by EFA6R or its mutants (EFA6RΔCC and EFA6RΔPH). COS7 cells were transfected with ARF6-HA and various amounts of GFP-EFA6R (*WT*), GFP-EFA6RΔCC (Δ*CC*), or GFP-EFA6RΔPH (Δ*PH*) plasmids. After 2 days, cells were serum-starved for 2 h and lysed, ARF6-GTP was precipitated from lysates with GST-GGA3 PBD beads, and the precipitates were then immunoblotted with anti-HA antibody. The transfected cells were also homogenized and centrifuged to obtain the cytosol and membrane fractions. The cell lysates and membrane fractions were immunoblotted with anti-HA and anti-GFP antibodies to determine expression levels of total ARF6 and EFA6R or its deletions mutants, respectively. Densitometric analysis of the ARF6-GTP is shown after normalization to the expression of EFA6 or its mutants in the total (*panel I*) or in the membrane fraction (*panel II*). **, *p* < 0.01; ***, *p* < 0.001; *ns*, not significant. *Error bars* represent S.D.

To resolve whether or not the PH and CC domains are required for the ARF6 GEF activity of EFA6R, the GST-GGA3 PBD pulldown assay was carried out after co-transfection of HA-ARF6 with various EFA6R deletion or point mutant constructs (Fig. 8*A*). We made a point mutant of EFA6R tagged with GFP in which a highly conserved glutamic acid was mutated to lysine (E134K) to render the protein catalytically inactive. These catalytically inactive mutants (E134K and ΔSec7) of EFA6R could not activate ARF6. The deletion mutants containing the functional Sec7 domain but lacking the PM targeting ability (Sec7 and ΔPH) also failed to activate ARF6. The EFA6RΔCC mutant, which showed weak PM localization, activated ARF6 but to a lesser extent when compared with EFA6R. When targeted to the PM via addition of the C*AAX* motif of K-Ras (which targets the proteins to membranes through its farnesylation), the Sec7 domain alone (Sec7-C*AAX*) was sufficient to induce the ARF6 activation. However, the Sec7 domain with a catalytically inactive point mutation (E156K) was unable to activate ARF6 following addition of the C*AAX* motif (Sec7_E134K_-C*AAX*). These results indicated that EFA6R requires not only the catalytic Sec7 domain but also PM localization through the PH and CC domains to act as a GEF for ARF6.

We then sought to determine whether the reduction in the ARF6 GEF activity of EFA6R by deletion of the CC domain was due to a reduction in the PM localization. To this end, we studied the dose-dependent effect of EFA6R and EFA6RΔCC on ARF6 activation. EFA6RΔPH, which does not activate ARF6, was used as a negative control in the assay. We have shown that the activation of ARF6 by EFA6R and EFA6RΔCC occurs in a dose-dependent manner as increasing the amount of EFA6R/EFA6RΔCC plasmid transfected into the cells with a constant amount of ARF6 increased the amount of GTP-bound ARF6 detectable in the GST-GGA3 PBD pulldown (Fig. 8*B*, *panel I*). At any given concentration, the amount of ARF6 activated by EFA6RΔCC was lower than that activated by EFA6R when the ARF6-GTP levels were normalized to the total EFA6R/EFA6RΔCC protein expression. However, the amount of ARF6 activated by EFA6RΔCC was indistinguishable from that activated by EFA6R at all concentrations when the ARF6-GTP levels were normalized to EFA6R/EFA6RΔCC protein expression in the membrane fraction (Fig. 8*B*, *panel II*). These data suggest that EFA6RΔCC is less active as an ARF6 GEF than EFA6R because of its weak PM localization. These data also confirm that EFA6R dissociation from the PM requires both PI 4,5-P_2_ depletion and actin destabilization.

##### EFA6R Co-localizes with ARF6 and Causes Reorganization of the Actin Cytoskeleton

**FIGURE 9.**
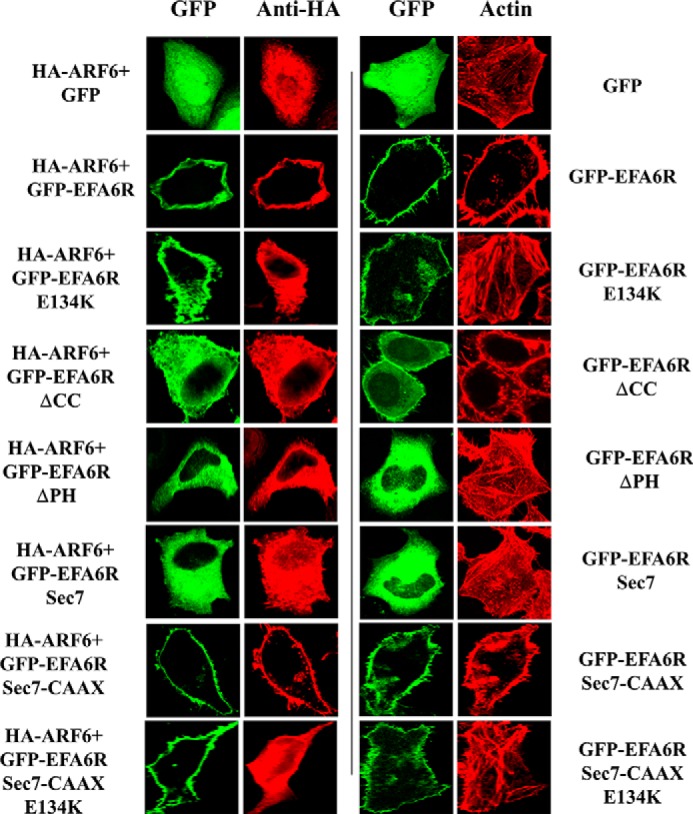
**Effect of EFA6R and its mutants on ARF6 localization and loss of actin stress fibers.** HeLa cells were transiently transfected with GFP, GFP-EFA6R, or its mutants with or without ARF6-HA. After 2 days, the cells were serum-starved, fixed with paraformaldehyde, immunostained with an anti-HA antibody or rhodamine-conjugated phalloidin (for actin), and imaged using a confocal microscope. The images are representative of 85–90% of 90–110 transfected cells from three different cell preparations.

ARF6 relocates from endosomes to the PM upon its activation and induces cortical actin formation at the PM and concomitant loss of actin stress fibers (24). To confirm that EFA6R can act as a GEF for ARF6 *in vivo*, we examined the effect of EFA6R and its mutants on the subcellular localization of ARF6 in HeLa cells (Fig. 9). For this purpose, we expressed GFP-tagged EFA6R or its point or deletion mutants together with HA-ARF6 in HeLa cells and assessed ARF6 localization by immunofluorescence using an anti-HA antibody. As observed previously (24), a punctate staining pattern was observed for ARF6 in HeLa cells (data not shown), and its localization was unaltered when co-expressed with control GFP. However, ARF6 was recruited to the PM in cells co-transfected with GFP-EFA6R and HA-ARF6. EFA6RΔCC, which showed weak PM localization, caused partial relocation of ARF6 (∼35% of total) to the PM in cells co-transfected with GFP-EFA6ΔCC and HA-ARF6. Neither the catalytically inactive mutant (E134K), which localized to the PM, nor the ΔPH and Sec7 deletion mutants, which did not localize to the PM but contained the active Sec7 domain of EFA6R, were able to induce ARF6 redistribution to the PM in the transfected cells. The PM-targeted Sec7-C*AAX* but not the Sec7_E134K_-C*AAX* mutant of EFA6R was able to cause ARF6 redistribution to the PM. These data confirm that EFA6R requires both its GEF activity and its association with the PM to activate ARF6.

We then examined the loss of actin stress fibers in cells expressing GFP-tagged EFA6R or its mutants as a readout of the ability of EFA6R to activate endogenous ARF6 (Fig. 9). The actin network in the control GFP-transfected cells was similar to that in untransfected cells (data not shown). GFP tagging had no effect on the actin network. The loss of actin stress fibers was observed in cells expressing GFP-EFA6R, suggesting that EFA6R induces loss of actin stress fibers. However, the E134K, ΔPH, and Sec7 mutants of EFA6R were unable to promote loss of actin stress fibers in the transfected cells. The EFA6ΔCC mutant induced partial loss of actin stress fibers, whereas Sec7-C*AAX* induced a loss of actin stress fibers, which was similar to that of EFA6R, and the catalytically inactive Sec7_E134K_-C*AAX* did not. This analysis also suggests that EFA6R-induced loss of actin stress fibers was dependent on both the PM recruitment and the GEF activity of EFA6R.

**FIGURE 10.**
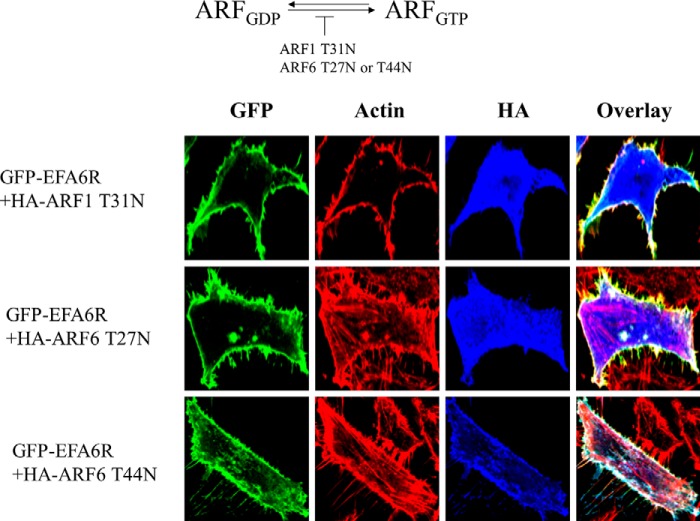
**The constitutively inactive mutant of ARF6, ARF6_T27N_ or ARF6_T44N_, prevents potentiation of actin stress fiber loss by EFA6R.** *Top*, a schematic representation showing the ARF mutants that locked in the inactive GDP-bound form. *Bottom*, HeLa cells were transiently transfected with either GFP or GFP-EFA6R with ARF6_T27N_-HA, ARF6_T44N_-HA, or ARF1_T31N_-HA. After 2 days, the cells were serum-starved, fixed with paraformaldehyde, immunostained with an anti-HA antibody and rhodamine-conjugated phalloidin (for actin), and imaged using a confocal microscope. The images are representative of 85–90% of 75–90 transfected cells from three different cell preparations.

To confirm that EFA6R induces loss of actin stress fibers by activating ARF6, we transfected HeLa cells with GFP-EFA6R and the HA-tagged constitutively inactive mutant of ARF6 (ARF6_T44N_ or ARF6_T27N_) or ARF1 (ARF1_T31N_) and analyzed loss of actin stress fibers. As shown in Fig. 10, the constitutively inactive mutants of ARF6, but not that of ARF1, reversed the loss of actin stress fibers induced by EFA6R. This result indicates that EFA6R causes the loss of actin stress fibers specifically through ARF6 activation.

## DISCUSSION

ARFs regulate membrane trafficking at multiple sites within the cell. Mammalian cells express six ARFs, over 15 ARF GEFs, and 24 ARF GTPase-activating proteins. Because the ARF GEFs and ARF GTPase-activating proteins outnumber ARFs, it has been suggested that the ARF regulators contribute to the site-specific regulation of ARFs. Therefore, understanding the ARF specificities and functions of these regulators is extremely useful in assessing spatiotemporal dynamics of ARF cellular functions. However, the ARF substrate specificity, localization, and functions are unknown for many of these regulators. In this study, we have demonstrated the ability of EFA6R, a new member of the EFA6 subfamily of ARF6 GEFs, to localize to the PM by binding PIP_2_ through the PH domain, to increase cellular levels of ARF6-GTP, and to stimulate reorganization of the actin cytoskeleton.

Because ARFs 1–5 are mainly found at the Golgi, whereas ARF6 localizes to the PM, it has been suggested that the substrate specificity of ARF GEFs is likely to be conferred by their subcellular localization. We have shown that EFA6R is constitutively localized to the PM, suggesting that it may act as a specific GEF for ARF6 *in vivo*. This hypothesis is also suggested by the binding of EFA6R to PI 4,5-P_2_ as PI 4,5-P_2_ has previously been implicated in the localization of EFA6A and several other proteins to the PM (42, 43). The requirement of both the CC and PH domains in membrane recruitment was assessed by using CC and PH deletion mutants of EFA6R and the compounds ionomycin and cytochalasin D. The loss of the CC domain or actin destabilization by cytochalasin D treatment abolished the PM localization of EFA6R upon depletion of PIP_2_ levels, indicating that the CC domain and intact actin cytoskeleton are important for a secondary site of association between EFA6R and the actin cytoskeleton/PM. Furthermore, EFA6R lacking the PH domain localized to the cytosol. Together these data suggest that binding of the PH domain to PIP_2_ is required for EFA6R localization to the PM, which is further stabilized by a secondary site of association between EFA6R and the actin cytoskeleton and/or plasma membrane. EFA6A in contrast has been shown to localize to the PM by binding both PIP_2_ and actin through the PH domain (39). However, EFA6A has also been shown to bind α-actinin, an actin-binding protein, through its C-terminal CC domain in dendritic spines (44). Unlike EFA6A, we found that EFA6R did not interact directly with F-actin *in vitro* (data not shown). Because the CC domain of EFA6R is highly homologous to that of EFA6A, it is tempting to speculate that EFA6R may also bind to actin cytoskeleton via α-actinin.

We confirmed the selectivity of EFA6R for ARF6 by co-expressing the proteins in an *in vivo* system and specifically precipitating out GTP-bound ARF from cell lysates. To do this, we took advantage of the interaction between ARFs and a family of ARF effectors, the GGA proteins, which bind to ARFs in a GTP-dependent manner (45), by expressing the ARF-binding VHS-GAT domain as a GST fusion protein. ARF6-GTP levels were increased upon co-expression with either EFA6R or EFA6A, a previously characterized GEF for ARF6 (15), whereas ARF1-GTP levels did not increase when expressed with either EFA6R or EFA6A but are increased when co-expressed with the ARF1 exchange factor BIG1 (46, 47). Furthermore, ARF6-GTP levels were decreased upon knockdown of EFA6R expression. These data suggest that EFA6R acts as an ARF6-specific GEF *in vivo* and that this activity occurs in a concentration-dependent manner as increasing the amount of EFA6R plasmid used for transfection of the cells increased the amount of ARF6-GTP in the lysates.

To determine the role of different domains of EFA6R on the activation of ARF6, we co-expressed ARF6 with various deletion constructs of EFA6R and repeated the GST-GGA3 PBD assay. Results indicate that the CC and PH domains are both required for membrane targeting because the Sec7 domain alone when artificially targeted to the PM with the C*AAX* motif from Ras was sufficient to stimulate ARF6-GTP binding to a level comparable with that of the full-length protein. We also found that expression of active but not catalytically inactive (E134K) EFA6R caused translocation of ARF6 from an intracellular, presumably endocytic, compartment to the PM, suggesting that ARF6 translocation is dependent on its bound nucleotide.

To investigate the functional relevance of EFA6R-mediated ARF6 activity, we examined the effect of overexpression of various EFA6R constructs on the morphology of the actin cytoskeleton. In agreement with previous studies suggesting that increased levels of ARF6-GTP cause rearrangements of actin (15, 23, 24, 47), we found that expression of EFA6R or its Sec7 domain targeted to the membrane by addition of the C*AAX* motif caused a loss of actin stress fibers. The expression of either the catalytically inactive EFA6R_E134K_ or any of the deletion mutants with abolished GEF activity toward ARF6 did not induce loss of actin stress fibers, suggesting that EFA6R causes a loss of actin stress fibers by activating ARF6. Consistent with this, EFA6R-induced actin rearrangements were reversed by the constitutively inactive mutant of ARF6 but not that of ARF1. Furthermore, EFA6R knockdown affected endogenous ARF6 activation. As mentioned in the Introduction, there is accumulating evidence for differential EFA6R expression in malignant diseases such as ovarian cancer (7, 14, 19–22), and experiments are now in progress to determine the role of EFA6R in ovarian cancer. In this regard, the findings reported in this study will assist further investigation of ARF6 GEF activity-dependent and -independent roles of EFA6R in ovarian cancer.

## Footnotes

2 The abbreviations used are:

ARF: ADP-ribosylation factor
EFA6: exchange factor for ARF6
HCA67: hepatocellular carcinoma-associated antigen 67
PSD3: pleckstrin and Sec7 domain-containing 3
PH: pleckstrin homology
CC: coiled coil
PM: plasma membrane
GEF: guanine nucleotide exchange factor
PI: phosphatidylinositol
P_2_: bisphosphate
PIP_2_: PI 4,5-bisphosphate
FKBP: FK506-binding protein 12
GGA3: Golgi-associated, γ-adaptin ear-containing, ARF-binding protein 3
TRITC: tetramethylrhodamine isothiocyanate
PBD: protein binding domain
CFP: cyan fluorescent protein
FRB: FKBP12-rapamycin binding
mRFP: monomeric red fluorescent protein
NHS: *N*-hydroxysuccinimide
PLC: phospholipase C.
